# Disposable platform for bacterial lysis and nucleic acid amplification based on a single USB-powered printed circuit board

**DOI:** 10.1371/journal.pone.0284424

**Published:** 2023-04-26

**Authors:** Kamal G. Shah, Mike Roller, Sujatha Kumar, Steven Bennett, Erin Heiniger, Katriel Looney, Joshua Buser, Joshua D. Bishop, Paul Yager

**Affiliations:** Department of Bioengineering, University of Washington, Seattle, Washington, United States of America; Rutgers Biomedical and Health Sciences, UNITED STATES

## Abstract

Recent advances in electronics and microfluidics have enabled several research groups to develop fully integrated, sample-to-result isothermal nucleic acid amplification test (NAAT) platforms for the point of care. However, high component counts and costs have limited translation of these platforms beyond the clinic to low-resource settings—including homes. Many NAATs include complex, multi-component heater electronics based on flex circuits or multiple printed circuit boards (PCBs) to support essential NAAT steps such as lysis, sample deactivation, and nucleic acid amplification. In contrast, current commercial assays for home use, such as those for pregnancy or ovulation that include electronics, typically have just one onboard PCB. This work describes a generalizable strategy to integrate all heaters and the electronics needed to control them onto a single low-cost, USB-powered PCB. We built a multiplexable disposable NAAT (“MD NAAT”) platform that applies these principles, integrating small-area heaters that heat small regions to near-boiling (for pathogen lysis and deactivation) and large-area heaters (for amplification) on the same PCB. We show that both classes of heaters have high intra-board and inter-device reproducibility despite only heating a NAAT cartridge from below. We validated the small-area heaters by lysing methicillin-resistant *Staphylococcus aureus* (MRSA) cells and the large-area heaters by performing two types of isothermal NAATs (isothermal strand displacement amplification (iSDA) and loop-mediated isothermal amplification (LAMP)). These results demonstrate the merit of integrating NAAT heaters and control electronics onto a single printed circuit board and are a step toward translating NAATs to the home.

## Introduction

Infectious diseases contributed to 6.3 million deaths globally in 2016, comprising 11.4% of total deaths [1]. Highly sensitive and specific diagnostic tests are needed to facilitate early diagnosis and treatment, especially at the point of care. Paper-based assays such as lateral flow tests are suitable for the point-of-care due to their low cost, rapid time-to-result, and minimal user steps, but they suffer from poor sensitivity [2–4]. In contrast, many nucleic acid amplification tests (NAATs) are highly sensitive and specific but require complex user or manufacturing steps and/or costly instruments [5], thereby relegating them to laboratory use. Combining the merits of both assay formats is amplifying nucleic acids in a paper-and-plastic point-of-care device isothermally and image with a mobile phone, as our lab first demonstrated in 2016 with the sample-to-result “MAD NAAT” (multiplexed autonomous disposable NAAT) platform [6].

Several recent publications describe microfluidic NAAT platforms [6–10]. Several steps in the NAAT workflow require heating, such as sample deactivation and nucleic acid amplification. Many NAAT platforms, therefore, include onboard chemical [11–14] or electrical [6–8] heaters. However, most of these platforms (especially devices integrating sample lysis) have large component counts and require complex assembly steps. For example, our lab’s MAD NAAT platform required 57 discrete components that were precisely aligned and soldered [6]. Other NAATs in the literature similarly rely on external heaters [9], flexible circuits [7], costly temperature sensors [7], Peltier elements [10], or multiple printed circuit boards [6] to precisely attain or maintain NAAT-relevant temperatures. In contrast, current commercial point-of-care tests intended for home use (e.g., disposable automatic digital pregnancy or ovulation tests such as the Clearblue™ digital) integrate all electronics onto a single rigid printed circuit board with surface-mounted components.

In this manuscript, we describe a strategy to substantially improve point-of-care NAAT platform manufacturability by integrating all power distribution, heater, communication, and control electronics onto a single, 2-layer rigid printed circuit board (PCB), which we call the “MD NAAT” (multiplexable disposable NAAT). The USB-powered PCB includes surface-mounted resistors for localized heating (e.g., lysis heating or valve actuation) and controlled-resistance wire traces for heating large areas uniformly (e.g., for amplification) in microfluidic cartridges. We describe a reproducible strategy to provide good thermal contact between the heater and other disposable elements while minimizing undesired heating of other device components. Finally, we show the platform’s utility to lyse methicillin-resistant *Staphylococcus aureus* (MRSA) bacteria and perform isothermal nucleic acid amplification for MRSA target genes in a porous matrix despite only heating a cartridge from below. The integrated heaters in the MD NAAT are a substantial step toward translating NAATs from laboratories to the home.

## Methods

### Device overview

**Fig 1:**
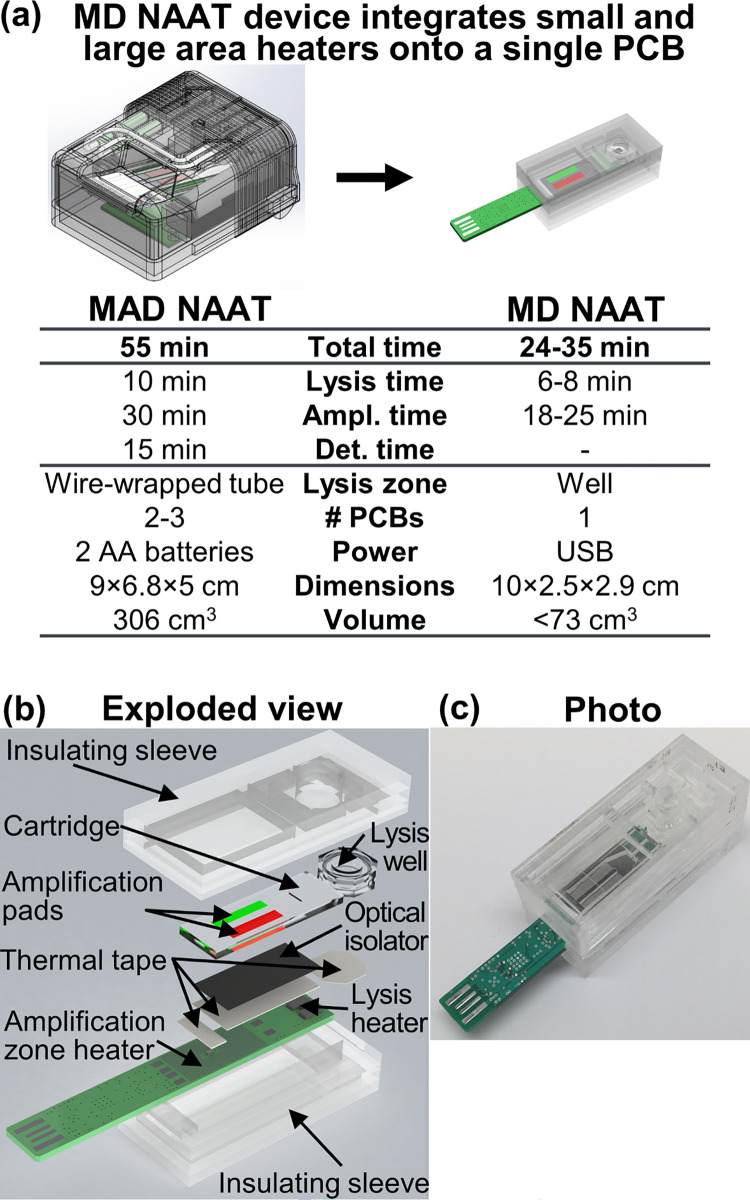
Summary of the multiplexed, disposable NAAT (MD NAAT) and its autonomous predecessor, the MAD NAAT. (a) The new MD NAAT platform reduces device complexity and size (by integrating all electronics on one PCB) and lowers the time to result. (b) The new MD NAAT device implements large-area (e.g., amplification zone) and small-area (e.g., lysis) heaters and their control electronics on a single 2-layer USB-powered PCB. (c) A photo of the MD NAAT device shows that the amplification pads are visible through the plastic insulation (i.e., compatible with real-time fluorescence or *in situ* optical detection) because the PCB heats the cartridge to lysis and amplification-relevant temperatures from below.

Fig 1A shows the MD NAAT device with integrated small and large-area heaters on a single USB-powered PCB for lysis and amplification, respectively (previously published battery-powered MAD NAAT device is shown for comparison [6]). The MD NAAT device consists of a rigid printed circuit board, a disposable paper and plastic cartridge with amplification reagents, and an outer sleeve to insulate the assay (Fig 1B). The printed circuit board contains regions with localized resistor-based heaters and a large area with a resistive wire trace heater. Thermally conductive tape (3M 8815) adheres to the printed circuit board heaters to the disposable cartridge. Note that in the development phase of this device, we have reused the PCBs but replaced all fluidic components after each use to prevent contamination from one test to the next. A commercial implementation can continue this practice with the PCB as part of a permanent instrument and the fluidic components as disposable. However, we consider that the PCB could be so inexpensive at the production scale that it could be permanently affixed to the fluidic components and be disposed of as a single unit after a single use, as with existing commercial pregnancy or ovulation tests. The amplification pads in the MD NAAT device are visible through the plastic insulation, allowing for real-time fluorescence measurement, thus reducing the detection time to 35 min from the 55 min required on the MAD NAAT device, which used an integrated lateral flow strip for detection.

#### Design of the insulated sleeve

For real-time fluorescence measurement of the NAAT assay, an optically clear, nonfluorescent insulated sleeve was designed by layering laser-cut, chemically welded 1/16” and 1/8” PMMA sheets (Fig 1C). Air voids were located above and below the temperature-regulated zone where nucleic acid amplification occurred to minimize conductive losses through the PMMA. A mobile phone (Nexus 5X) fluorescence reader, detailed in Shah *et al*. [15], was fixed 15 cm above the insulated sleeve to balance the competing needs of maximizing the field of view while ensuring a sufficient numerical aperture to resolve fluorophores at amplification-relevant concentrations.

#### Design of the NAAT cartridge and electronics

The disposable cartridge consisted of a PMMA base and glass fiber membranes in which nucleic acid amplification occurred (Fig 1B). The 1.59 mm thick base layer of the PMMA cartridge contained two 0.45 mm deep laser-etched regions into which 3×15 mm glass fiber membranes (GE Standard 17) were placed (about 20 µL capacity). The cartridge also contained an upstream lysis well (about 300 µL volume) with two additional laser-cut PMMA layers bonded with dichloromethane (~3.6 mm thick total). The cartridge top was sealed with clear adhesive tape (Bio-Rad MSB1001), while the bottom of the amplification zone was optically isolated from the printed circuit board with a 0.1 mm-thick sheet of black low-density polyethylene (LDPE, McMaster-Carr). Adhesive-coated thermally conductive tape (3M 8810) provided thermal contact between the LDPE to the printed circuit board (PCB).

The PCB was a standard 2-layer board made of FR-4 (150°C glass transition temperature), was powered by a universal serial bus (USB, 5V at up to 500 mA), and contained a microcontroller and USB interface bridge (Cypress Semiconductor CY8C4245LQI-483 and CY7C65211-24LTXI). S1 Fig shows a schematic of the PCB

The design of the PCB layers is illustrated in S2 Fig.

The S1 Table indicates a comprehensive bill of materials for the PCB.

**Fig 2:**
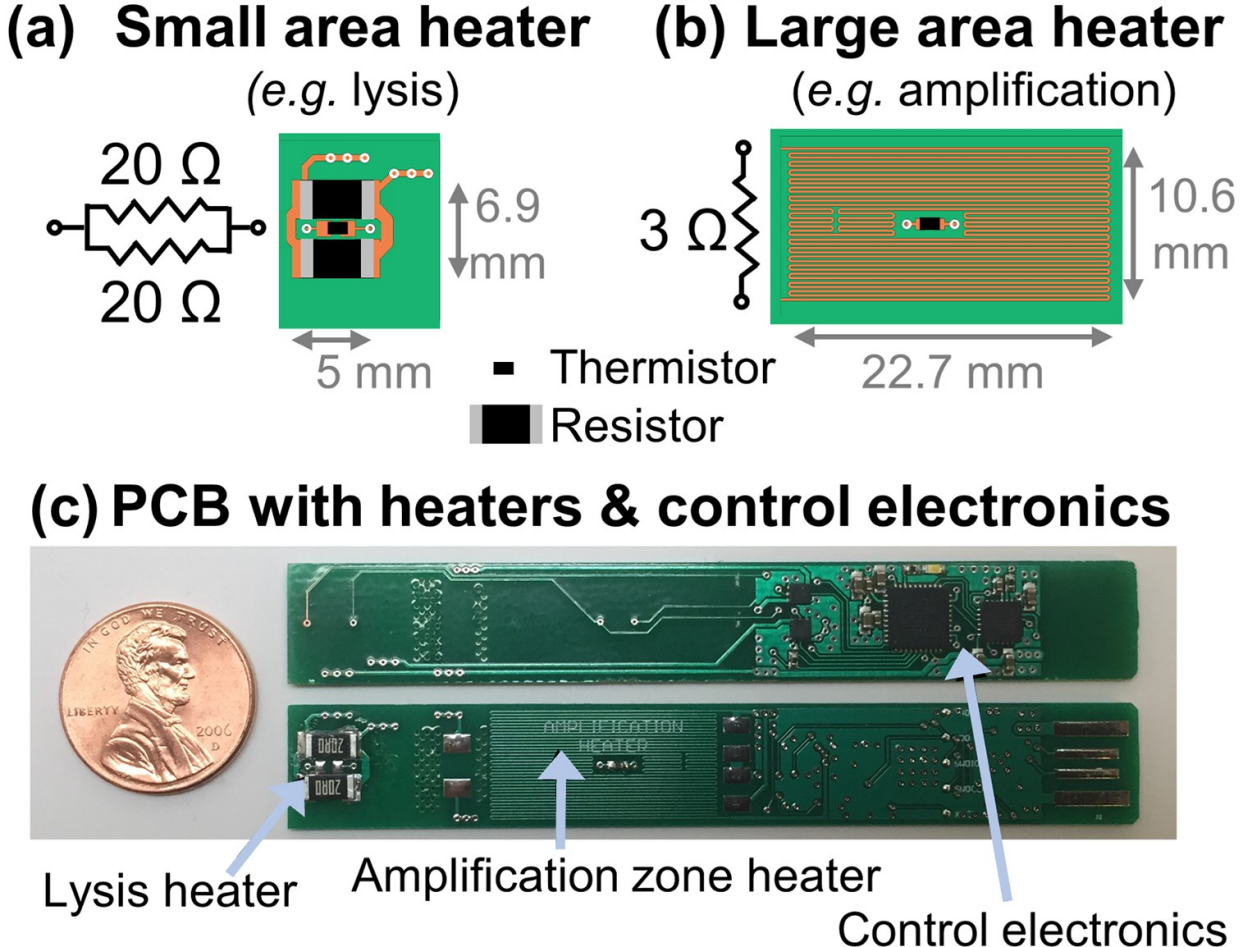
Diagrams of the two types of heaters on the PCB. (a) The small-area heaters consisted of surface-mounted resistors in parallel (the heater can be made smaller using a smaller resistor package). (b) The large-area heater consisted of a serpentine copper trace in the PCB itself. The temperatures of both types of heaters can be actively regulated with a thermistor via proportional-integral control. (c) A photo of the top and bottom of the MD NAAT PCB implements both heater types (bottom layer) and control electronics (top layer) on just one PCB, with a US penny for scale. The MD NAAT PCB includes areas for optional post-lysis or post-amplification valves, but these are not used here.

The details of small and large-area heaters and control electronics on the PCB are given in Fig 2. The small-area heaters consisted of surface-mounted resistors in parallel (Fig 2A). The lysis zone was heated by passing a current through two resistors (20 Ω, ¾ W each); an optional NTC thermistor could be used to actively regulate the temperature with PID control if necessary. The large area heater consisted of a serpentine copper trace in the PCB itself (Fig 2B). Amplification zone temperatures were actively regulated to 55.3°C with proportional-integral control (Kp = 65535, Ki = 10) by passing a current through a serpentine trace (nominal 3 Ω resistance) and monitoring an NTC thermistor (Panasonic ERT-J0EG103FA) in the middle of the amplification zone at 5 Hz. The PCB was fabricated by Sunstone Circuits (Mulino, OR) using default manufacturing parameters (two-layer board, 1 oz. copper).

### Thermal validation

The thermal performance of the small-area heaters was evaluated by assessing the heaters’ ability to heat water to near-boiling temperatures from below. Four sets of insulating sleeves and printed circuit boards were fabricated and evaluated on two separate days with three cartridges (i.e., replicates) each day. The PMMA cartridges were adhered to PCBs with thermal tape, lysis wells were filled with 200 µL of water (>18.0 MΩ•cm), the heaters were powered for 8.5 minutes in an open loop configuration, and liquid temperatures were monitored with T-type thermocouples for ten minutes at 1 Hz.

Given the lower thermal mass and target temperatures of the amplification zone compared to the lysis zone, we monitored the temperature of the thermistor (at 5 Hz) during NAAT runs on two sets of insulating sleeves and PCBs and nine cartridges (i.e., replicates) per day over two days. Statistical analysis consisted of performing two-way ANOVAs on the ramp-up time to threshold temperatures. All data analysis was performed in Microsoft Excel 365 and MATLAB r2019a.

### Nucleic acid amplification chemistry

An isothermal strand displacement amplification (iSDA) method for the methicillin-resistant *Staphylococcus aureus*(MRSA) *ldh1* and *mecA* gene targets, and an internal amplification control (IAC) previously described was used [6, 16]. The IAC template used was a synthetic double-stranded DNA that is flanked by primer binding sites that matched the *ldh1/mecA* target primer sequence and detected by its interior-sequence-matching probe. The probe for IAC is labeled with a different fluorophore than the *ldh1*/*mecA* target detection probe. Nucleic acid amplification was performed in a Whatman Standard 17 glass fiber membrane, as detailed in Lafleur *et al*. and Kumar *et al*. [6, 17]. The membranes were typically laser cut, blocked in 1% BSA + 0.1% Tween-20 for 1 hour, and dried at 45°C in an incubator at least overnight before use. Each pad contained 18 µL of iSDA master mix as described previously; briefly, the chemistry consisted of: inorganic potassium phosphate buffer (pH 7.6, 50 mM), magnesium sulfate (5.63 mM), an equimolar mix of deoxynucleotides [dATP, dCTP, dGTP, dTTP] (0.4 mM), trehalose (0.27 M), 500 kD dextran (4.8% (w/v)), DNA primers (500 nM forward, 250 nM reverse, 50 mM of each bumper primers), target-specific fluorescent probes (minor groove binders) labeled with a Texas Red analog (AquaPhluor-593, 200 nM), internal amplification control-specific probes labeled with 6-FAM (200 nM), Bst 2.0 Warmstart DNA polymerase (New England BioLabs, 300 U/µL), Nt.BbvC1 nicking enzyme (New England BioLabs, 2.4% (v/v)), and nuclease-free water. The trehalose and dextran aided enzyme preservation and controlled rehydration [17]. Nucleic acid sequences of primers, probes, and DNA templates are published in Lafleur *et al*. [6]. The master mix was pipetted onto blocked pads, flash-frozen with liquid nitrogen, lyophilized overnight, and stored in heat-sealed, aluminized Mylar pouches at room temperature with desiccant until use as described in Kumar *et al*. [17].

### Performing the NAAT

NAAT runs on the MD NAAT device consisted of placing the PCB and cartridge assembly in the insulated PMMA sleeve, powering via USB, and performing bacterial cell lysis and/or nucleic acid amplification. MRSA bacteria (ATCC BAA-1556) was grown to mid-log phase and suspended in 10 mM Tris buffer (pH 8.0). Ten thousand MRSA cells (100 µL) were added to the lysis well of a cartridge along with 100 µL of achromopeptidase (ACP) enzymes (Sigma A3547, 3.0 U/µL in 10 mM Tris), and the mixture was incubated for two minutes. The lysate was thermally deactivated in the MD NAAT by heating for 4 minutes with a small-area heater unless otherwise indicated.

For amplification reactions, two glass fiber pads containing lyophilized iSDA reagents were placed in the cartridge and were rehydrated with 20 µL of MRSA lysate (for MRSA-positive), 10^5^ copies of IAC DNA (for positive controls), or 10 mM Tris buffer (for true negatives). The cartridges were then sealed, and the PCB was powered by USB and heated the amplification zone with the large-area heaters for 45 minutes. As detailed in Shah *et al*. [15], a mobile phone fluorescence reader imaged the amplification zone in time-lapse mode using the Android Camera FV-5 application (0.25 second exposure, ISO 3200, incandescent white balance, about 30 seconds between frames, flash on). The mobile phone reader was equipped with dual-bandpass fluorescence excitation (over the flash) and emission (on the camera) filters for fluorescein and Texas Red (Semrock Brightline dual-band bandpass filter). Amplification reactions were also performed in a plate reader (Molecular Device SpectraMax iD3) by mounting glass fiber pads containing lyophilized iSDA reagents onto a clear PMMA plate which was laser cut to the size of a 384-well plate. The glass fiber pads were positioned to correspond to 4 wells of a 384-well plate. The plate reader heated the trays to 50.5°C and imaged the pads in fluorescence kinetic mode (593 nm excitation, 650 nm emission, 1 s exposure). All analysis was performed in MATLAB R2019a. Details of imaging and signal processing have been published previously [18]. The description for loop-mediated amplification in the MD NAAT platform is provided in S1 Text.

To calculate the lysis efficiency, MRSA cells (5 x 10^4^, 1 x 10^5,^ and 2 x 10^5^) and achromopeptidase were added to the lysis well of the MD NAAT cartridge or a 1.5 ml microfuge tube placed in a heat block set to 95°C and lysed as described above. The volume of lysates from the MD NAAT cartridge was measured for any fluid loss due to evaporation. Quantitative PCR (qPCR) using Bioline SensiFAST™ SYBR® qPCR master mix (Thomas Scientific) with primers against the *ldh1* gene (reported in Heiniger et al. [19]) was used to quantify DNA recovery after lysis from the cartridge and the microfuge tube control. The starting quantity of MRSA DNA was measured against a standard curve of quantified MRSA DNA (ATCC BAA_1556). The total DNA copies recovered were calculated by multiplying DNA copies per microliter as reported by qPCR by the total lysate volume recovered. Results were normalized to the microfuge tube-heat block condition, and the percentage lysis efficiency was calculated.

## Results

### Thermal validation

**Fig 3:**
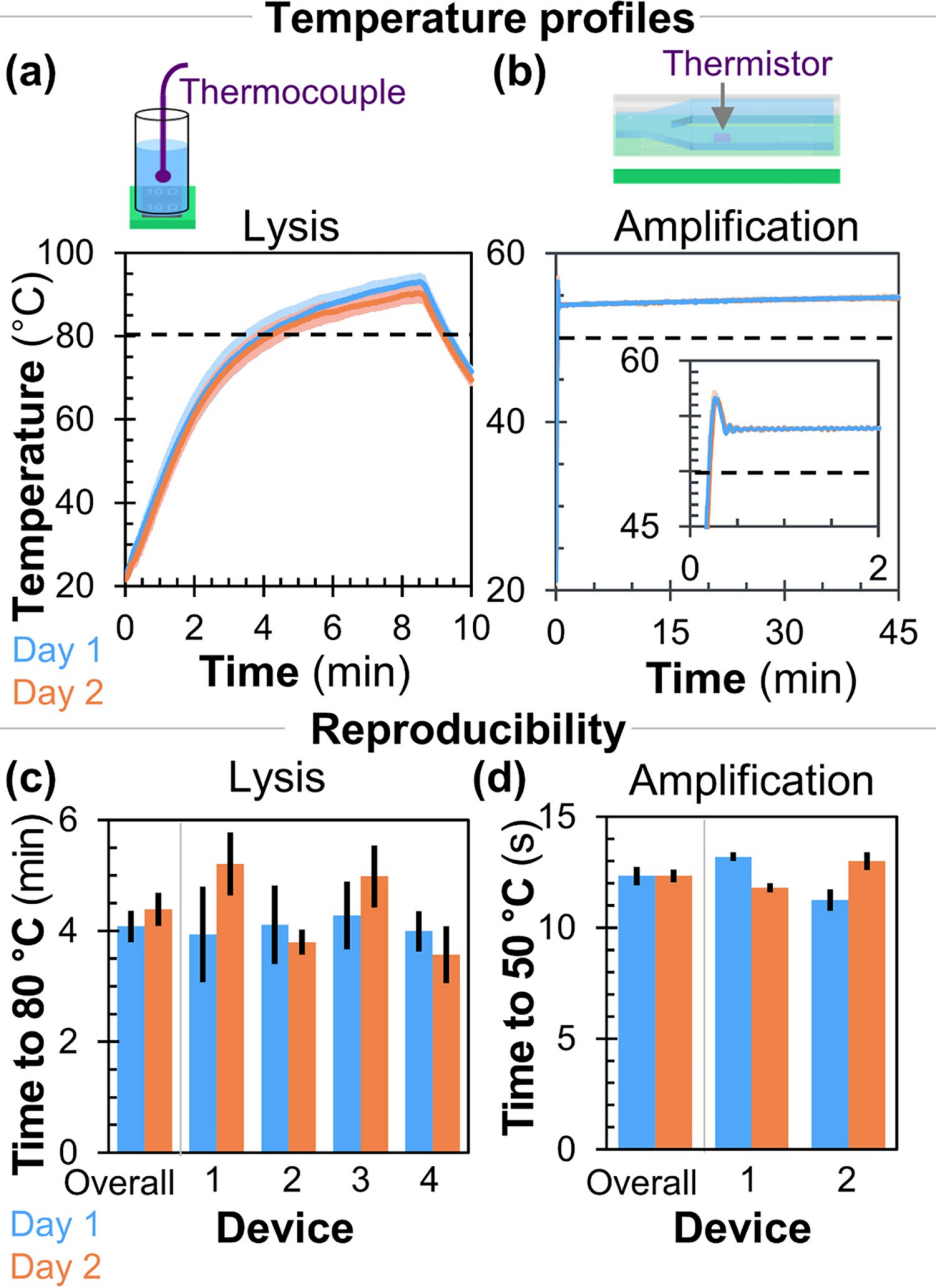
Reproducibility of small-area and large-area heaters between multiple devices and days. (a) Temperature profiles of a thermocouple in a liquid-filled well above a small-area heater that was powered for 8.5 minutes were reproducible between days (curves show average and 95% confidence intervals of data from four devices with three replicates each). (b) Temperature profiles of a thermistor in the large-area heater during nucleic acid amplification runs also showed substantial reproducibility (curves showed average and 95% confidence intervals from nine runs on two devices). Panels c and d show ramp-up times (time to 80°C (c) and 50°C (d)) for the profiles in panels a and b; bars indicate the mean and standard error of the mean. There was no significant difference in ramp-up time between days or groups for both types of heaters (p>0.05, two-way ANOVA).

We initially validated the thermal performance of the localized and large-area heaters on the printed circuit board. In both experiments, laser-cut PMMA cartridges with flat-bottomed surfaces were adhered to the PCB with thermally conductive tape. First, we assessed whether surface-mounted resistors could heat a liquid-filled well to near-boiling temperatures reproducibly from below, which is a common operation in NAATs to lyse pathogens or deactivate lytic enzymes. Two 20 Ω resistors (¾ W) were powered in parallel with about 500 mA total in an open-loop configuration for 8.5 minutes. Fig 3A shows temperature-time profiles of thermocouples in wells filled with 200 µL of water. The liquid temperature reached 80°C in about 4 minutes on four devices (n = 3) on two days (Fig 3C). The temperature continued to increase to about 90°C while the resistors were powered (i.e., until 8.5 minutes). The liquid was above 80°C for about 5 minutes on average. Fig 3C shows no significant difference in the ramp-up time to 80°C between devices or days at the 5% significance level (respectively: p = 0.37 and p = 0.46, two-way ANOVA).

Second, we assessed the ability of the large-area wire trace heaters to attain NAAT-relevant temperatures on two devices on two days. The wire traces were supplied with 5V under proportional-integral control (set point 55.3°C). We actively monitored the temperature of the NTC thermistor protruding from the middle of the amplification zone at 5 Hz. Fig 3B shows that the amplification zone reached NAAT-relevant temperatures with a slight 1.4°C overshoot at 20 seconds relative to the set point of 55.3°C on day 1 (n = 9); similar results were observed on day 2. All devices reached 50°C within 13 seconds. The temperature of the amplification zone was 53.97±0.26°C (mean ± 95% confidence interval) at 30 seconds and rose slightly to 54.77±0.30°C at 45 minutes. As before, there was no significant difference in the ramp-up time to 50°C between devices or days at the 5% significant level (Fig 3D, p = 0.57, and p = 0.82, two-way ANOVA). Thermocouple measurements of liquid-filled cartridge temperatures suggest a ramp-up time to iSDA temperatures (>48.5°C) of about 3 minutes.

### Applying small-area heaters to lyse bacteria

On-chip cell lysis is a challenging step in point-of-care NAATs. One strategy to lyse gram-negative bacteria that we have shown with great success [6, 12, 19, 20] is to treat samples with an enzyme mixture such as an achromopeptidase (ACP) crude extract, followed by thermal deactivation of the enzyme mixture by heating to near-boiling temperatures. Lysis at near-boiling temperatures is challenging because the amplification reagents (especially the heat-sensitive enzymes) are stored near the lysis chamber and must remain near room temperature prior to introduction of the lysed sample. We, therefore, minimized undesired heating of the amplification zone during the lysis heating step by using separate pieces of thermally conductive tape in each heating area and placing an array of holes in the PCB between the two zones. To study the heat transfer between the lysis chamber and the amplification zones, we placed thermocouples in the lysis chamber and the amplification zone PMMA cartridge, and measured temperature in the two zones during lysis. While the lysis chamber temperature reached 95°C, the amplification zone temperatures remained close to the ambient temperatures for 8 minutes (S3 Fig).

**Fig 4:**
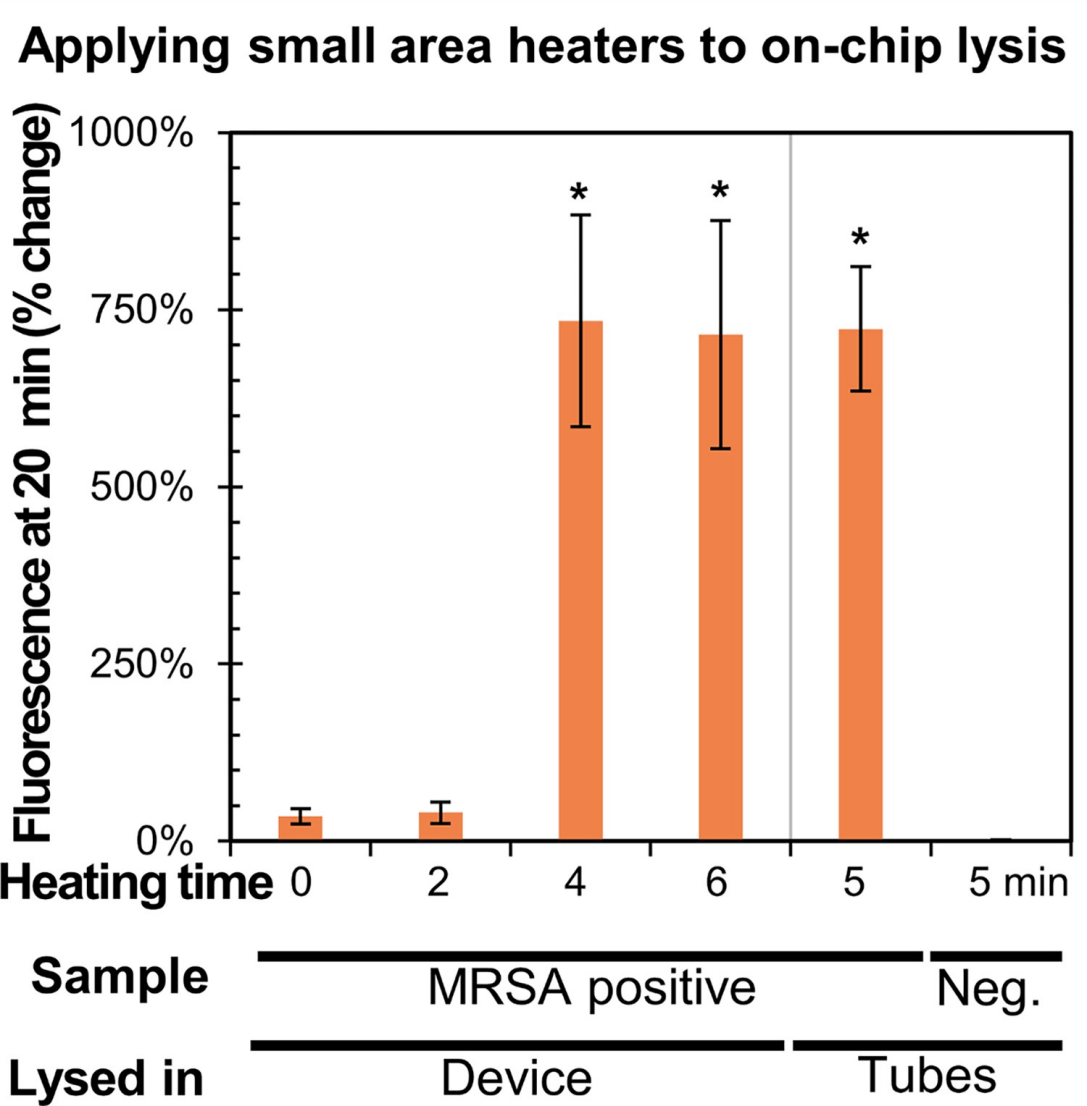
Small-area heaters lyse bacteria on the chip. MRSA cells were lysed with achromopeptidase enzymes and thermally deactivated in tube controls (n = 3 tubes) in a heat block or with the small-area heaters in the MD NAAT (n = 3 devices). Twenty µL of each lysate rehydrated lyophilized pad containing reagents for isothermal strand displacement amplification (iSDA), placed in a PMMA tray, heated to 50.5°C, and imaged in a plate reader in fluorescence mode in real-time. Cell suspensions heated in the lysis chamber for at least 4 minutes showed significantly higher MRSA-specific fluorescence than negative controls after 20 minutes of amplification (* p<0.00001, one-way ANOVA). Bars show mean and error bars show standard error of the mean.

First, we validated that the MD NAAT device could produce lysate containing amplifiable DNA. We applied the localized resistive heaters to lyse methicillin-resistant *Staphylococcus aureus* (MRSA) grown to the mid-log phase. About 90,000 MRSA cells were added to the flat-bottomed PMMA cartridges in 3 devices, co-incubated with ACP for 2 minutes, and thermally deactivated at a range of heating times. We assessed lysis effectiveness by using the lysate to rehydrate glass fiber pads containing lyophilized nucleic acid amplification reagents, which were then incubated in a plate reader at 50.5°C and imaged in fluorescence mode. Real-time amplification curves showed that MRSA lysate heated with the small-area heaters for four or six minutes in the MD NAAT amplified similarly to lysate from in-tube positive controls (Fig 4). For these samples, the average fluorescence at 20 minutes was significantly greater than that of a negative control lacking MRSA cells that had been heated in tubes (p<0.0001, one-way ANOVA). These tube-heated negative controls that were heated in tubes did not amplify.

Next, we calculated the lysis efficiency of the small-area heater compared to in-tube lysis in a heat block using MRSA cells (5 x 10^4^, 1 x 10^5,^ and 2 x 10^5^) and quantified by qPCR. We found ~40% lysis efficiency compared to the in-tube lysis. We found that there was considerable fluid loss to evaporation in the open-topped cartridge compared to the in-tube lysis control in the heating block.

### Applying both small and large heaters to a real-time fluorescence NAAT

Finally, we tested the small-area heaters for lysis in conjunction with the large-area wire trace heaters for amplification on the same MD NAAT platform. We validated the heaters with fluorescence assays for MRSA using NAATs that contained sequence-specific fluorescent probes for MRSA (red-emitting or internal-amplification control (IAC, green-emitting) [6, 16]. While the assays could be run simultaneously in biplexed reactions (e.g., when the IAC is a colocalized positive control), this section focuses on the use of heaters to support isothermal NAATs such as isothermal strand displacement amplification (iSDA) and loop-mediated amplification (LAMP).

**Fig 5:**
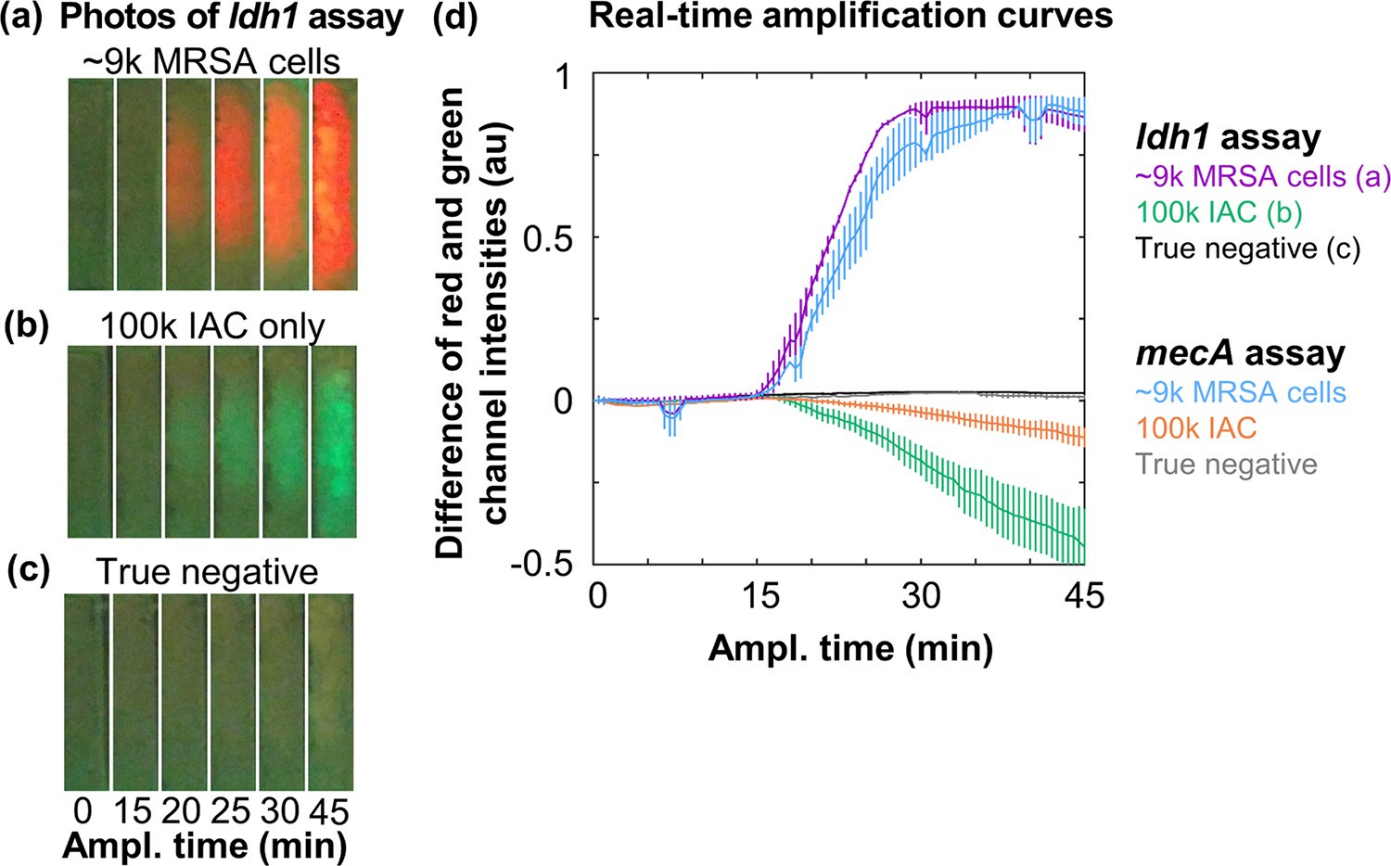
On-chip lysis and amplification with two assays (*ldh1* and *mecA* genes) in the MD NAAT. MRSA cells were lysed in the MD NAAT device with ACP for two minutes and thermally deactivated for four minutes by heating to ~95°C. The lysate was then pipetted to rehydrate amplification (ampl.) pads containing lyophilized iSDA reagents. The pads were heated by the MD NAAT (the x-axes indicate when the amplification heaters were powered on). (a-c) Mobile phone images of pads show no amplification in the true negatives, substantial green fluorescence in pads containing only internal amplification control (IAC) DNA, and substantial red fluorescence in pads containing MRSA cells for the *ldh1* assay. Similar results were observed for the *mecA* assay. (d) Real-time curves show high signal-to-noise ratios and rapid amplification. On average, MRSA-positives showed measurable amplification within 18 minutes (p<0.001 for *ldh1* and p<0.01 for *mecA* relative to respective true negative controls, one-way ANOVA). Curves show mean and standard error of the mean for n = 3 pads (IAC only and true negatives) or n = 2 (MRSA-positive samples); positive signals (i.e., redder images) indicate target-specific amplification, while negative signals (i.e. greener images) indicate IAC-specific amplification.

MRSA cells were grown to the mid-log phase, lysed in the MD NAAT device with ACP for two minutes, and thermally deactivated all the enzymes by heating to ~95°C for four minutes. The deactivated lysate was then pipetted out to rehydrate downstream glass fiber pads containing lyophilized NAAT reagents. The large-area heater provided the required temperature for the NAAT reactions, which were imaged in real-time with the previously described two-fluorophore mobile phone reader (for green and red-emitting fluorophores). Fig 5 shows images and real-time amplification curves for pads specific to the *ldh1* or *mecA* genes (both present in MRSA). Lysate containing MRSA (about 9000 copies per pad) showed substantial red fluorescence within 20 minutes for the *ldh1* (Fig 5A) or *mecA* genes when imaged with a Nexus 5X phone. Pads containing 100k copies of internal amplification control DNA had strong green fluorescence in about 25 minutes for both assays (Fig 5B). Truly negative samples (lacking MRSA and IAC) showed neither red nor green fluorescence during the entire 45 minutes of amplification time (Fig 5C). The real-time curves in Fig 5D showed similar liftoff times for both assays: the fluorescence at 18 minutes was significantly higher in MRSA-positives than in the negative controls for both the *ldh1* (p<0.0001, one-way ANOVA) and *mecA* (p<0.01, one-way ANOVA) assays. Similar amplification times (~20 minutes to liftoff) were observed in loop-mediated isothermal amplification (LAMP) performed with genomic DNA in buffer (S4 Fig).

## Discussion

NAAT devices intended for the point of care must integrate several steps that require heating to high temperatures (e.g., ~95°C to lyse samples or actuate valves [6, 21]) or to specific temperatures with tight tolerances (e.g., ~50 or ~64°C for nucleic acid amplification). In this work, ~95°C was needed at the lysis step to break the MRSA bacterial cell wall, deactivate achromopeptidase enzymes that would otherwise destroy amplification enzymes downstream, and fragment the genomic DNA [6, 19]. We implemented iSDA and LAMP amplification methods that operated at ~50°C and ~64°C, respectively. This manuscript describes a strategy to implement two types of heaters targeted to these distinct heating requirements on a single, two-layer rigid printed circuit board, thermal validation of the heaters, and application to bacterial lysis and amplification on the MD NAAT platform.

Many NAATs intended for the point of care are based on paper microfluidics, but these NAATs must overcome paper’s high surface-area-to-volume ratio and low thermal mass, which substantially complicate uniform heating. Approaches to uniformly heating include incorporating materials with high thermal conductivity [9] high thermal mass [6, 22], or phase change at the target temperature (often coupled to exothermic chemical reactions) [6, 23]. However, these strategies often suffer from high component counts and are not amenable to integration onto a single rigid printed circuit board [6, 8]. In contrast, the large-area heater described in this work requires just one extra component (<$0.15 thermistor) coupled to a serpentine wire trace in the PCB. This approach compares favorably with recent work that used costly infrared temperature sensors (>$40) to monitor the temperature of an area heated by a serpentine trace in a flexible circuit [7].

However, serpentine traces are not suitable for heating small areas due to the PCB trace’s low electrical resistivity, which cannot dissipate sufficient heat at a particular location. Heating small areas rapidly with discrete, surface-mounted resistors addresses this limitation and provides the added benefit of precisely defining the heater area with deliberate resistor package selection. One drawback of integrating this type of small-area heater with a wire-trace large-area heater onto a single PCB is poor thermal contact between the PCB and NAAT cartridge. This is because the upper surfaces of the two types of heaters are not coplanar (i.e., the resistors nominally protrude 0.6 mm and the thermistor 0.5 mm relative to the wire traces in the PCB itself).

Therefore, we explored strategies to planarize the PCB surface relative to the bottom of the NAAT cartridge. We evaluated whether a given strategy addressed the competing needs of rapid/uniform heating, low board-to-board variability, PCB reuse during development, and ease of manufacturing at both laboratory and larger scale volumes. We found that thermally conductive double-sided adhesive met these needs while ensuring consistent thermal performance between boards and days (Fig 3), which was not true for other adhesives such as CNC-machined planar thermal epoxy (which introduces irregularities from degassing, is labor-intensive, and is poorly reproducible) or polydimethylsiloxane (PDMS) tape. Thermally conductive double-sided tape is especially useful in this application because it is available at specific thicknesses (near the nominal resistor thicknesses) and conforms to surface deformities, which ensures that the tape accommodates manufacturing defects, thermistors or other components protruding from the PCB surface. Adhering the bottom of the cartridge to just one PCB with thermally conductive tape for each heater zone enabled us to perform common NAAT operations such as lysis in wells and amplification in the paper (Fig 5). We showed minimal heat transfer between the lysis chamber and the amplification zone during lysis at 95°C and ensured that the temperature rise observed caused no damage to the dry reagents stored in the adjacent amplification zone (S3 Fig). However, we encountered significant fluid loss during heat lysis at 95°C with variability in the volumes recovered. Future iterations of the MD NAAT device will involve designs to minimize evaporation.

The MD NAAT, which contains a printed circuit board with integrated small and large-area heaters, improves upon other heater designs in the NAAT literature. Many recent NAATs are heated by laminates, such as wire traces in flex circuits or positive temperature coefficient (PTC) resistive heaters [7, 8, 24], but these suffer from high component counts, costly sensors, and limited ability to scale the area heated. Almost all NAATs that lyse samples and amplify nucleic acids on chip require separate (or external) heaters for the two NAAT steps (or costly components). In contrast, the heaters described in this paper are integrated onto a single PCB and only heat the NAAT cartridge from below, which substantially improves manufacturability and enables real-time fluorescence detection on the MD NAAT. The cost for the PCB components listed in S1 Table is for a laboratory prototype, and mass production will substantially reduce the cost per device (roughly estimated to be 10-fold). While the components were assembled individually, assembly can be automated during mass production.

The PCB also has a pre-amplification zone valve heater with one resistor and a post-amplification zone valve heater with two resistors in parallel (S1 Fig). A heat-activated valve design between the lysis and amplification zones can be programmed to open with heat and aid fluid flow from the lysis zone to the amplification zone. While in this study, we directly pipetted the lysate from the lysis zone into the amplification pad with lyophilized reagents, a heat-activated valve design can be incorporated into an integrated sample-to-result MD NAAT, which can perform the full assay without user intervention. Similarly, the post-amplification valve heater can be activated for MD NAAT platforms with integrated lateral flow detection instead of real-time fluorescence detection.

The MD NAAT platform can be used for any biological specimen containing pathogens requiring heat lysis and can be integrated with any isothermal amplification method. However, each biological sample is unique, requiring specific chemical treatment to minimize inhibitors such as mucus, RNases, and DNases that may adversely affect the detection limit or interfere with the fluorescence signal. For blood and urine, upstream processing fixtures to remove blood cells or to filter samples can be integrated into the MD NAAT device. We adapted the MD NAAT platform with a filtration device for pathogen concentration and demonstrated heat lysis, valve activation, and DNA amplification using urine spiked with *Chlamydia trachomatis*-infected cells as a proof-of-principle sample-to-result integrated device [25].

To summarize, this manuscript describes an evolution of the MAD NAAT device that incorporates the first integrated PCB that 1) heats both small and large areas for NAATs; 2) heats to a precise temperature reproducibly between devices; 3) is easily powered by consumer-grade electronics (USB 2.0 at 5V with maximum 500 mA current draw); 4) can be manufactured at default PCB fabrication conditions; 5) implements standard NAAT operations such as lysis and amplification on just one printed circuit board.

## Conclusions

We have demonstrated a generalizable approach to integrating small and large-area heaters onto a single, USB-powered rigid printed circuit board using default PCB manufacturer fabrication parameters. The heaters enable on-chip bacterial lysis and nucleic acid amplification on the MD NAAT platform and are a step toward translating NAATs to the home. In a recent publication using the MD NAAT platform, we have shown near-digital amplification with improved detection limits of MRSA genomic DNA between 100 and 316 copies in a biplexed reaction and reduced time-to-result between 15–20 minutes [18]. Future work from our group will report on demonstration of MD NAAT incorporating complex samples such as nasal swab and whole blood to detect viral RNA in fully integrated sample-to-result POC devices.

## Supporting information

S1 Fig Printed circuit board schematic shows the functional elements of the MD NAAT PCB with the electronic components used to implement them. (TIF)

S2 Fig Top (a) and bottom (b) views of the USB-powered printed circuit board underlying the MD NAAT. The board implemented all heaters on the bottom layer. The board includes heating zones for: lysis (far right), pre-amplification valves (not used), nucleic acid amplification (center), and post-amplification valves (not used).
(TIF)

S3 Fig Measurement of heat transfer between lysis chamber and amplification zone in the MD NAAT device. (a) Photo of the MD NAAT device with thermocouple (TC) placements in the lysis chamber and amplification zone. (b) Graph showing temperatures in the lysis chamber, the amplification zone PMMA, and PCB (thermistor) during lysis. While the temperature rises to 95°C in the lysis chamber, the adjacent amplification zones remain close to ambient (below 30°C).
(TIF)

S4 Fig Photos and real-time curves of loop-mediated amplification (LAMP) performed in the MD NAAT targeting the mecA gene of MRSA show the platform’s ability to perform LAMP (~64°C amplification temperature). (a) Photos of glass fiber pads during amplification show detectable amplification by about 25 minutes. The pads appeared fluorescent at time zero due to using an intercalating dye that labels all double-stranded DNA (*i*.*e*., the primers and templates present before the pads reach LAMP-relevant temperatures). (b) Real-time amplification curves in the green color channel show substantial amplification of MRSA-positive samples beyond 21 minutes (p<0.05 at 21 minutes and p<0.001 beyond 40 minutes relative to negative control, *t*-test, n = 3). Bars show the mean, and error bars show the standard error of the mean. (c) Sequences and concentrations of primers used in LAMP reactions.
(TIF)

S1 Table Printed circuit board heater bill of materials. (PDF)

S1 Text Protocol for loop-mediated amplification (LAMP) performed in MD NAAT device. Devices were prepared similarly to those for which isothermal strand displacement amplification was performed, except as noted below. BSA-blocked glass fiber pads were placed in laser-cut PMMA cartridges (as before) and were rehydrated with 20 µL of fresh sample mixed with LAMP master mix. The sample/master mix solution consisted of 10 µL of NEB WarmStart® LAMP Kit (DNA & RNA) (E1700S)), 0.4 µL of NEB LAMP Fluorescent Dye (Lot 10046978), 1 µL of primers (S4C Fig), water or 10^4^ copies of MRSA genomic DNA (ATCC BAA-1556DQ, strain FPR3757), and nuclease-free water to bring the volume to 20 µL. The cartridges were sealed and heated in the MD NAAT amplification zone for at least fifty minutes (set temperature 77°C, K_P_ = 65535, K_I_ = 0, K_d_ = 0). A Nexus 5X mobile phone with multipass excitation and emission filters for fluorescein and Texas Red imaged the pads in real-time about every 30 seconds as before (ISO 800, 0.25 second exposure, incandescent white balance). Images were analyzed in MATLAB 2019a.
(PDF)
