# Supplementary figures and images for: Disposable platform for bacterial lysis and nucleic acid amplification based on a single USB-powered printed circuit board
Source: PLoS One. 2023 Apr 26;18(4):e0284424. doi: 10.1371/journal.pone.0284424 (PMC10132542; doi:10.1371/journal.pone.0284424)

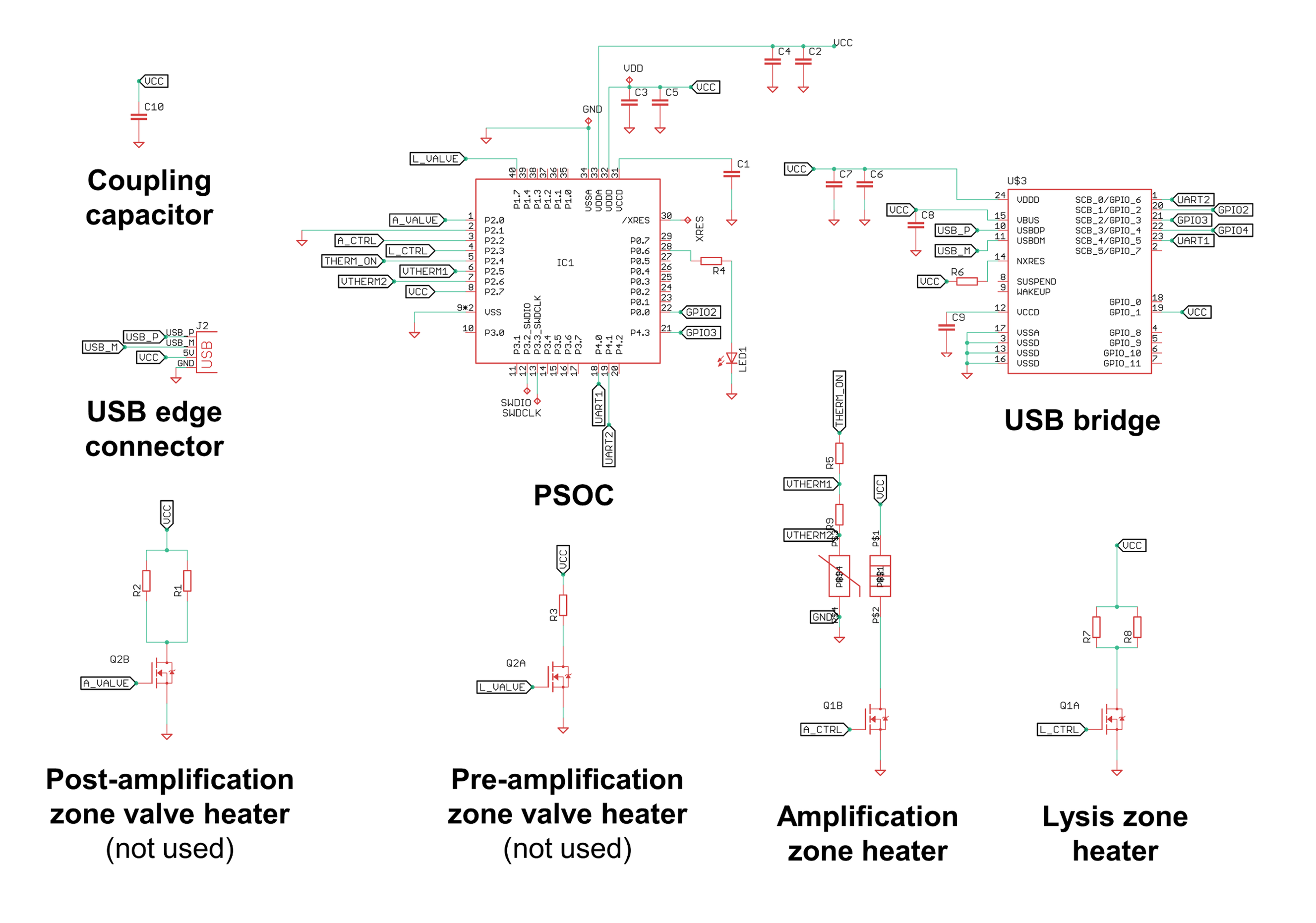

Supplement: S1 Fig — (TIF) [file pone.0284424.s001.tif]

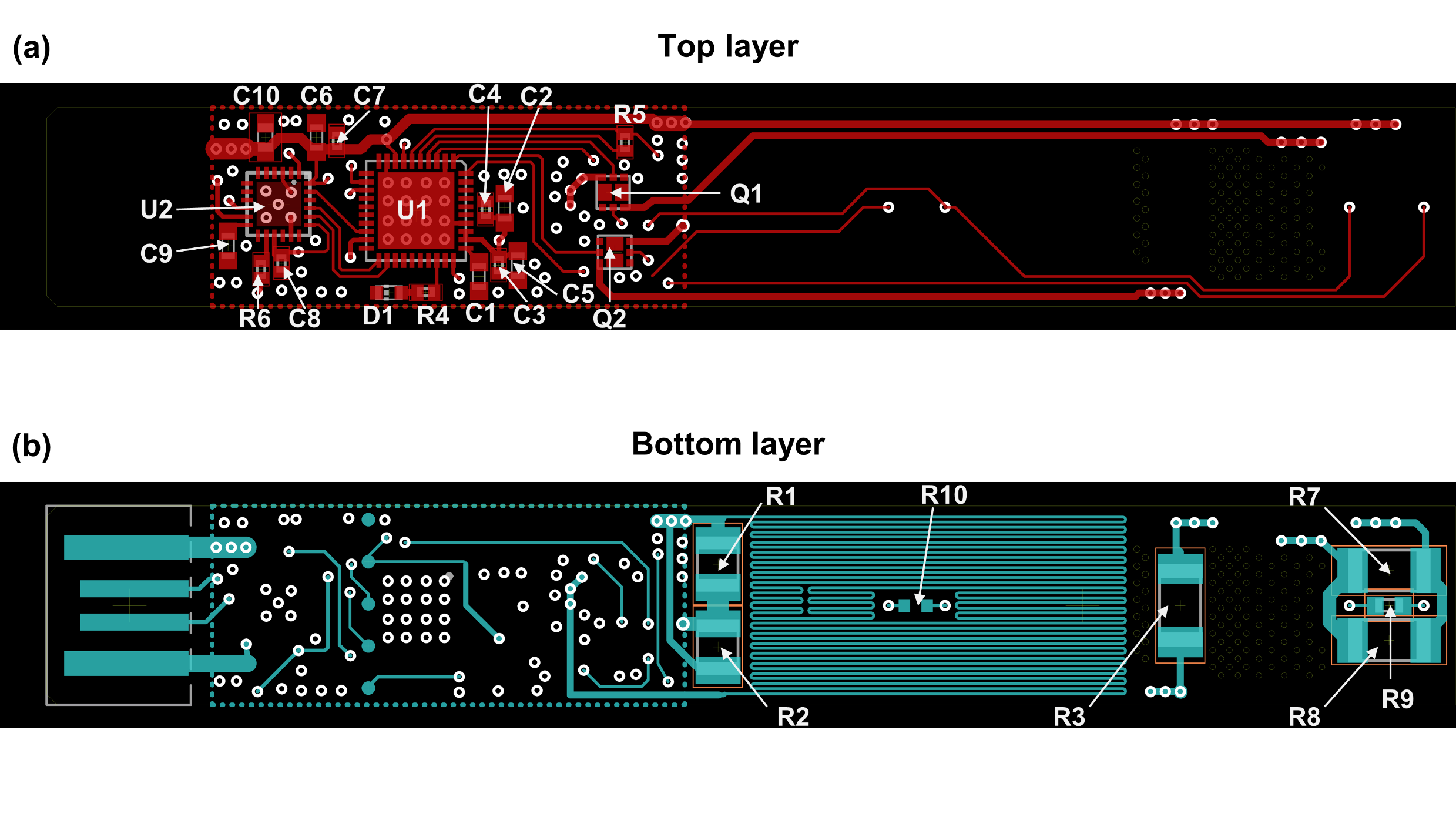

Supplement: S2 Fig — Top (a) and bottom (b) views of the USB-powered printed circuit board underlying the MD NAAT. The board implemented all heaters on the bottom layer. The board includes heating zones for: lysis (far right), pre-amplification valves (not used), nucleic acid amplification (center), and post-amplification valves (not used). (TIF) [file pone.0284424.s002.tif]

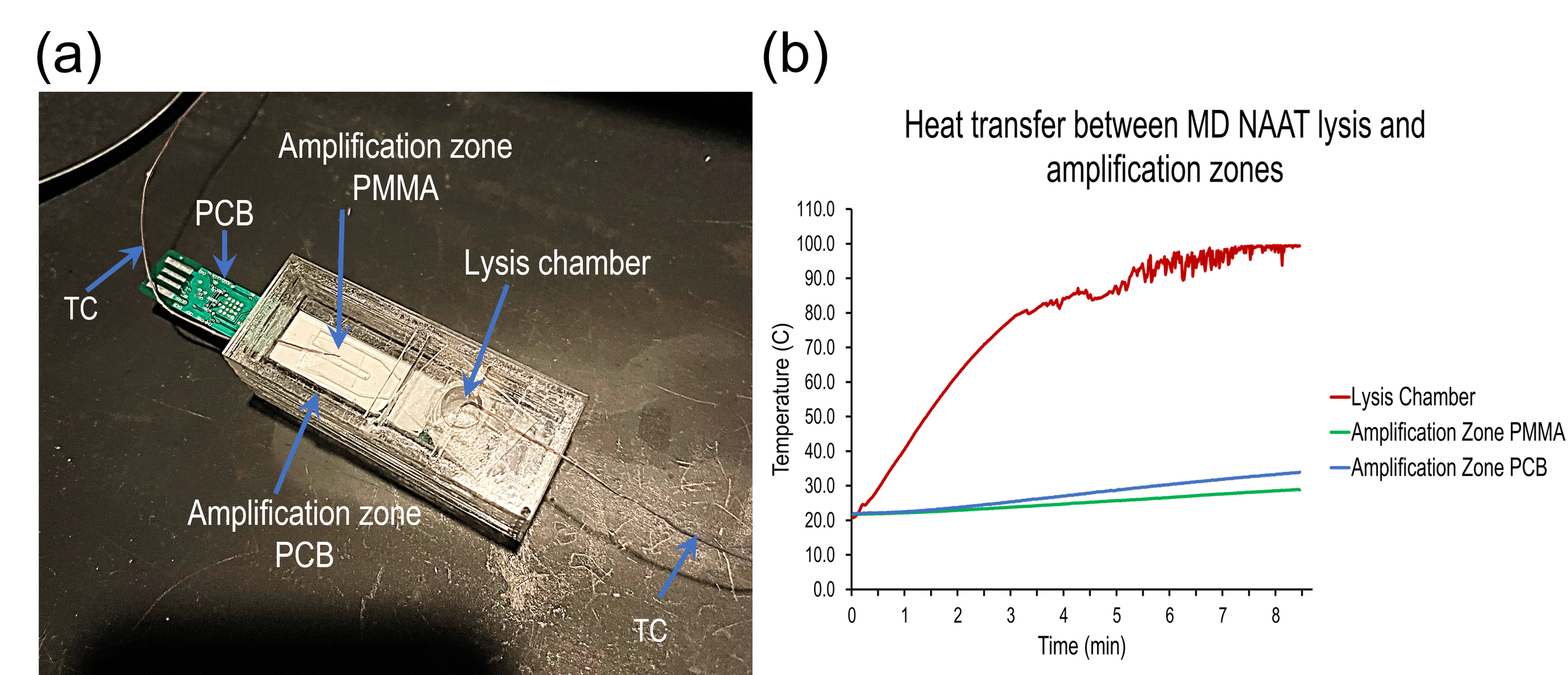

Supplement: S3 Fig — (a) Photo of the MD NAAT device with thermocouple (TC) placements in the lysis chamber and amplification zone. (b) Graph showing temperatures in the lysis chamber, the amplification zone PMMA, and PCB (thermistor) during lysis. While the temperature rises to 95°C in the lysis chamber, the adjacent amplification zones remain close to ambient (below 30°C). (TIF) [file pone.0284424.s003.tif]

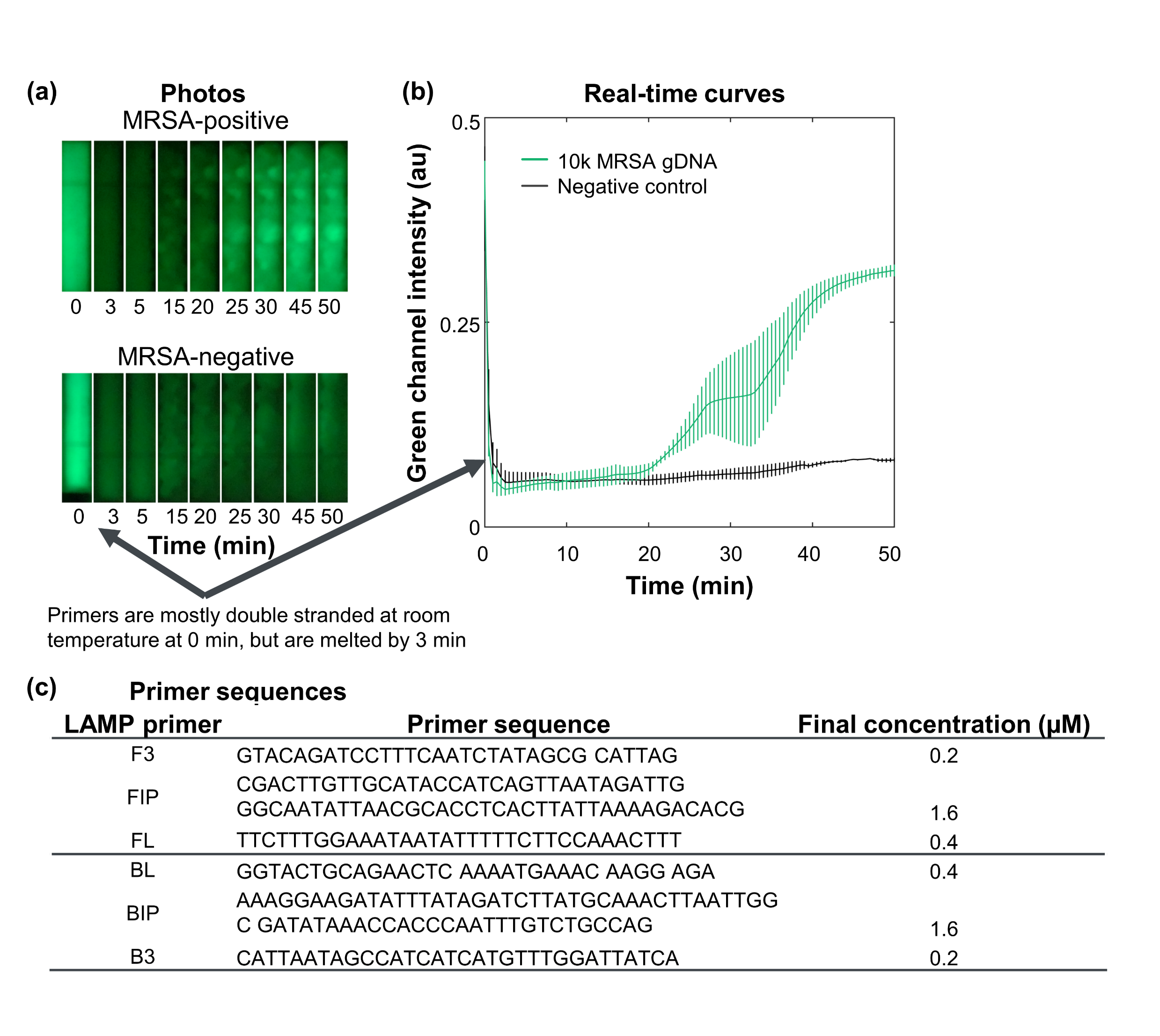

Supplement: S4 Fig — (a) Photos of glass fiber pads during amplification show detectable amplification by about 25 minutes. The pads appeared fluorescent at time zero due to using an intercalating dye that labels all double-stranded DNA (i.e., the primers and templates present before the pads reach LAMP-relevant temperatures). (b) Real-time amplification curves in the green color channel show substantial amplification of MRSA-positive samples beyond 21 minutes (p<0.05 at 21 minutes and p<0.001 beyond 40 minutes relative to negative control, t-test, n = 3). Bars show the mean, and error bars show the standard error of the mean. (c) Sequences and concentrations of primers used in LAMP reactions. (TIF) [file pone.0284424.s004.tif]
