# Supplementary material for: Disposable platform for bacterial lysis and nucleic acid amplification based on a single USB-powered printed circuit board
Source: PLoS One. 2023 Apr 26;18(4):e0284424. doi: 10.1371/journal.pone.0284424 (PMC10132542; doi:10.1371/journal.pone.0284424)
Supplement: S1 Table — (PDF) [file pone.0284424.s005.pdf]

S1 Table. Printed circuit board heater bill of materials

	Component (value, package*)	Distributor	Manufacturer	Part number	Quantity	Unit price†	Reference
1	MOSFET (6-VDFN)	Digikey	Vishay	FDMA1024NZ	2	$0.5733	Q1, Q2
2‡	PSOC (32 KB flash 4 KB SRAM, 40-QFN)	Digikey	Cypress	CY8C4245LQI-483	1	$2.9835	U1
3‡	USB bridge (24-QFN)	Digikey	Cypress	CY7C65211-24LTXI	1	$2.262	U2
4	White LED (0603)	Digikey	Osram	LW Q38E-Q1OO-3K6L-1	1	$0.204	D1
5	Resistor (360 Ω, 0402)	Digikey	Panasonic	ERJ-2RKF3600X	1	$0.0183	R4
6	Capacitor (100 nF, 0402)	Digikey	Murata	GRM155R71C104KA88D	4	$0.0152	C3, C4, C7, C8
7	Capacitor (1000 nF, 0603)	Digikey	Murata	GRM188R61A105KA61D	6	$0.039	C1, C2, C5, C6, C9
8	Capacitor (4700 nF, 0603)	Digikey	Kemet	C0603C475K9PACTU	1	$0.0565	C10
9	Resistor (4700 Ω, 0402)	Digikey	Panasonic	ERJ-2RKF4701X	1	$0.0225	R6
10	Resistor (10 Ω, 1210)	Digikey	Panasonic	ERJ-14YJ100U	2	$0.0551	R1, R2
11	Resistor (10 Ω, 2010)	Digikey	Panasonic	CRCW201010R0FKEF	1	$0.0993	R3
12	Resistor (20 Ω, 2010)	Digikey	Panasonic	CRCW201020R0FKEF	2	$0.0993	R7, R8
13	NTC thermistor (10 kΩ, 0402)	Digikey	Panasonic	ERT-J0EG103FA	2	$0.0839	R9, R10
14	PCB	Sunstone				$3.51	
	**Total**					**$11.07**†	

*Package sizes are imperial sizes

†Prices are at laboratory scale (100 units)

‡Components that only needed for laboratory development
