# Supplementary material for: Disposable platform for bacterial lysis and nucleic acid amplification based on a single USB-powered printed circuit board
Source: PLoS One. 2023 Apr 26;18(4):e0284424. doi: 10.1371/journal.pone.0284424 (PMC10132542; doi:10.1371/journal.pone.0284424)
Supplement: S1 Text — Devices were prepared similarly to those for which isothermal strand displacement amplification was performed, except as noted below. BSA-blocked glass fiber pads were placed in laser-cut PMMA cartridges (as before) and were rehydrated with 20 µL of fresh sample mixed with LAMP master mix. The sample/master mix solution consisted of 10 µL of NEB WarmStart® LAMP Kit (DNA & RNA) (E1700S)), 0.4 µL of NEB LAMP Fluorescent Dye (Lot 10046978), 1 µL of primers (S4C Fig), water or 104 copies of MRSA genomic DNA (ATCC BAA-1556DQ, strain FPR3757), and nuclease-free water to bring the volume to 20 µL. The cartridges were sealed and heated in the MD NAAT amplification zone for at least fifty minutes (set temperature 77°C, KP = 65535, KI = 0, Kd = 0). A Nexus 5X mobile phone with multipass excitation and emission filters for fluorescein and Texas Red imaged the pads in real-time about every 30 seconds as before (ISO 800, 0.25 second exposure, incandescent white balance). Images were analyzed in MATLAB 2019a. (PDF) [file pone.0284424.s006.pdf]

S1 Text. Protocol for loop-mediated amplification (LAMP) performed in MD NAAT device.

Devices were prepared similarly to those for which isothermal strand displacement amplification was performed except as noted below. BSA-blocked glass fiber pads were placed in laser-cut PMMA cartridges (as before) and were rehydrated with 20 µL of fresh sample mixed with LAMP master mix. The sample/master mix solution consisted of 10 µL of NEB WarmStart® LAMP Kit (DNA & RNA) (E1700S)), 0.4 µL of NEB LAMP Fluorescent Dye (Lot 10046978), 1 µL of primers (Figure S4c), water or 10^4 copies of MRSA genomic DNA (ATCC BAA-1556DQ, strain FPR3757), and nuclease-free water to bring the volume to 20 µL. The cartridges were sealed and heated in the MD NAAT amplification zone for at least fifty minutes (set temperature 77 °C, K_P=65535, K_I=0, K_d=0). A Nexus 5X mobile phone with multipass excitation and emission filters for fluorescein and Texas Red imaged the pads in real time about every 30 seconds as before (ISO 800, 0.25 second exposure, incandescent white balance). Images were analyzed in MATLAB 2019a.
